# Impact of wind speed and soil frost on electricity distribution system reliability

**DOI:** 10.1016/j.heliyon.2024.e40846

**Published:** 2024-12-04

**Authors:** Juha Haakana, Otto Räisänen, Markku Karhunen, Ilona Láng-Ritter, Jukka Lassila

**Affiliations:** a LUT University, Finland; b Finnish Environmental Institute, Finland; c Finnish Meteorological Institute, Finland

**Keywords:** Distribution system, Fault, Interruption, Power system, Reliability, Soil frost, Wind gust, Wind speed

## Abstract

Wind is a significant cause of power outages in overhead line networks. Present electricity distribution network management systems provide an extensive amount of data on network faults. These data combined with the openly available weather and soil information allow to analyze the impact of wind and soil frost on electricity system reliability more accurately than in the past. The objective is to determine the effect of soil frost on wind-induced faults in the electricity distribution system and identify variables that provide the best correlation between wind speed and the number of power supply interruptions. Five geographical areas in Finland are analyzed based on extensive electricity distribution interruption statistics collected in the country between 2008 and 2018. We introduce a methodology to analyze the effect of wind speed and soil frost and present numerical data analyzed for the areas under consideration. The methodology employs a multivariate linear regression model to show the dependence between wind, soil frost, and power supply interruptions. The results indicate that there is a strong positive correlation between the number of power supply interruptions and wind speed, and furthermore, in the multivariate model, adding the variable of soil frost improves correlation in all the areas under consideration. On average, the correlation with wind gusts and the number of power supply interruptions is approximately 0.67. However, with the soil frost variable, the correlation increases to 0.72. The results show that soil frost should be considered in studies on interruption modeling in Nordic conditions.

## Highlights

• A methodology using linear regression models to analyze the effects of windstorms and soil frost is presented.
• The statistical significance of soil frost on interruptions in power networks is shown with a multivariate linear model.
• The effect of soil frost on wind-induced power system interruptions is determined.

## 1 Introduction

Strong winds and windstorms can cause significant damage to society. For instance, power outages are one of the most common and important consequences of windstorms [1]. Finnish statistics show that one-third of all customer-experienced supply interruptions are caused by wind and storms. Other significant sources of interruptions are unknown causes, snow and ice, and component failures, accounting for 18 %, 14 %, and 11 % of interruptions, respectively. If we consider the time of interruptions experienced by end users instead of the number of interruptions, the proportion of wind-induced supply interruptions is even higher, up to 50 % [2].

Considering that today's society is highly dependent on a continuous supply of power, hardening of power lines and preparedness for windstorms are crucial in mitigating the socioeconomic impacts of power outages ([3]; [4]). Additionally, climate change has a significant impact on infrastructures of society [5]. Electricity distribution infrastructure is one of the sectors that is already suffering from climate change. Global warming itself is not directly critical to the electricity infrastructure, but its impacts can be seen, for instance, in increased costs caused by extreme weather events, such as storms and higher snow loads [6,7].

It is well known that a significant number of interruptions in the electricity distribution system are due to strong winds, windstorms [8,9], and snow loads affecting the overhead line network [2]. For instance, it is estimated that in the United States, approximately 80%–90 % of the power outages are due to extreme weather events [10]. In general, fault events causing interruptions of supply occur typically in network sections where falling trees may damage the power system. Laurila et al. [11] studied windstorms in Northern Europe (including Finland) and found that the wind gusts of cold-season windstorms are of the largest spatial scale, and the strongest wind gusts are recorded during the cold season (October–March). Laapas et al. [12] also found that the annual maximum wind speeds in Finland are typically observed in winter.

Most of the wind-induced power outages are due to the damage caused by uprooting or breaking trees falling on electricity distribution or transmission lines [13]. Finland is among the most forested countries in Europe, forests covering approximately 70 % of the area of Finland [14]. Furthermore, a large proportion of power lines especially in Finnish rural areas are located above ground and in forests. Information on the topic is available from the Energy Authority of Finland (EA) and the National Land Survey of Finland (NLS). For instance, in 2020, the proportion of overhead power distribution lines below 70 kV was 52 % [15], and in 2019, the proportion of overhead lines in the proximity of forests was 78 % (NLS, 2019). The figure contains all the lines surrounded by forest and the lines located close to single trees.

Seasonally freezing ground is a common phenomenon in areas with sufficiently long periods of below-freezing temperatures. The main factors affecting the duration and depth of soil frost are air temperature, soil type, vegetation, precipitation, and snow cover [16]. In the cold season, soil frost is typically present and anchors the trees to the ground thus making them more resistant against strong wind gusts [14,17,18].

### 1.1 Effect of climate change

Depending on location, climate change can have a varying effect on distribution network reliability. For instance, in northern locations, such as Finland, the climate is warming even faster than in the rest of the world. A recent study by Rantanen et al. [19] showed that in the Arctic region the warming has been at least four times as fast in the past 43 years (1979–2021) as the global average. In Finland in particular, the warming has been 2–3 times as fast as globally on average [19]. In a warmer climate, the depth of soil frost will decrease [20], and the period of soil frost is likely to become shorter in Finland [21]. Lehtonen et al. [21] state that the winter soil frost period will become roughly one month shorter by the 2050s, and the Finnish winters will have only short periods of soil frost. This could lead to an increase in wind-induced faults in the winter period.

Regarding windstorms, there is no clear consensus about how climate change affects them, nor about how the windiness will change in Finland. Ruosteenoja et al. (2022) produced model-derived climate projections for Finland by using results of the CMIP6 global climate model (Phase 6 of the Coupled Model Intercomparison Project, [22]), which is the newest generation of global climate models (GCMs). According to Ruosteenoja et al. (2022), the projected changes in the mean wind speed are minor, and by the 2080s changes in monthly mean wind speeds will vary between −12 and + 10 %. Kjellström [23], for instance, also states that there is no clear trend in the future in the occurrence of strong winds in the Nordic countries. Even though the changes in the occurrence of strong winds are small, compound events, such as strong wind and heavy precipitation simultaneously can increase the risk of extensive damage to critical infrastructure [24,25]. Gregow et al. [26] state that in Finnish forests the wind-induced risks will increase because of the lack of soil frost in the future. Additionally, several studies agree that although extratropical cyclones do not seem to amplify on average, the most extreme windstorms may get stronger in the future (Sinclair et al., 2020; Priestley and Catto, 2022). Especially in the southern and middle regions of Finland, the risk of wind damage will increase due to the shorter and milder soil frost period [14,21].

### 1.2 Effect of wind and soil frost on power system interruptions

Numerous studies have been made about wind risks to the forests in Finland and other Nordic countries [[26], [27], [28], [29], [30], [31]], and some studies have focused on the impacts of soil frost and winds on forestry [21,26,29,32].

The effect of wind on power supply interruptions has been investigated in a multitude of studies considering a wide variety of variables. However, there is a paucity of research related to the risks and impacts of soil frost or the combined effect of soil frost and wind-induced power supply interruptions on the electrical grid. The effect of soil has previously been considered on studies [[33], [34], [35],^36^] that have identified the anchoring effect of the soil during extreme events as a contributing factor for wind-induced interruptions. However, these studies have focused on the phenomenon of increased soil moisture reducing the anchoring effect and have not considered soil frost. Notably in the study [37] a large set of 218 variables were considered however soil temperature or soil frost was not included in the study.

Table 1 presents an overview of recent literature covering the research on the effect of wind and soil frost on electrical power system interruptions. The table shows that wind is the key element in several studies, but soil frost has not been addressed. Thus, this study, by combining the effects of both wind and soil frost, bridges a research gap.

**Table 1:** Key characteristics of the datasets used in studies on electricity network interruption analysis. A hyphen indicates information not disclosed or missing in the paper. The last row indicates the characteristics of this paper. OMS = outage management system, VMS = vegetation management system.

Publication year, reference	Analysis period	Data types non-DSO	Soil data types	Data types,DSO	Outage event types	Area size
[38]	1996.1998, 1999	Weather, soil,forest	Soil drainage	Infrastructure, OMS	Hurricane	2 DSOs
[39]	2000–2004	Population	–	Infrastrucutre, VMS	–	1 DSO, 764k customers, 28.6k km overhead line
[40]	1998–2004	Landcover, soil, weather	Soil drainage, soil depth	Infrastructure, OMS	Hurricane and ice storm	3 DSOs, 5.7M customers
[41]	1997–2005	Landcover, soil, weather	Soil moisture	Infrastructure, OMS	Hurricane	1 DSO, 125 000 km^2^
[42]	1997–2005	Landcover, soil, weather	Soil moisture	Infrastructure, OMS	Hurricane	59 700 km^2^
[43]	1997–2005	Landcover, soil, weather	Soil moisture	Customer count, infrastructure, OMS	Hurricane	59 700 km^2^
[44]	1997, 1998, 2004–2005	Landcover, soil, weather	Soil moisture	Customer count, infrastructure, OMS	Hurricane	78 500 km^2^
[45]	2005–2014	Landcover, soil, weather	Soil moisture	OMS, infrastructure	Blizzard, hurricane,nor'easter, thunderstorm	1 DSO, 1.2M customers, 29K km overhead line
[46]	2011	Forest, landcover, weather	–	Infrastructure, OMS, VMS	Hurricane	4600 km^2^
[33]	2005–2014	Landcover, soil, weather	Soil moisture	Infrastructure, OMS	Blizzard, hurricane,nor'easter, thunderstorm	1 DSO
[34]	1995–2005	Forest, landcover, population, topography, weather	Soil moisture	OMS	Hurricane	1 DSO
[35]	2007–2019	Landcover, vegetation, weather	Soil moisture	Infrastructure, OMS	Blizzard, hurricane,nor'easter, thunderstorm	1 DSO
[35]	2005–2017	Landcover, soil, weather	Soil moisture	Infastructure, OMS,VMS	Extratopical, thunderstorm	
[47]	2005–2018	Landcover, topography, vegetation, weather	Soil depth, soil moisture, soil type	Infrastrucutre, OMS	Nor'easter, snow and ice events	1 DSO
[8]	2010–2018	Forest, weather	–	Customer count, infrastructure, OMS	Extratopical	2 DSOs
[48]	2010–2016	Forest, weather	–	Infrastructure, OMS	Blizzard, extratropical, snow and ice events	1 DSO, 16 385 km^2^, 1.2M customers
[37]		Landcover, soil, weather, forest, vegetation	Soil moisture	Infrastructure, OMS	Extratropical and tropical storms	40,000 km^2^, 3.5M customers
[49]	2017–2019	Forest, topography, weather	.	Risk locations	Snow events	1 DSO, 9.2K overhead line, 80k customers
[9]	2005–2014	Weather	–	Customer count, infrastructure, OMS	Windstorm, thunderstorm	3.3M customers, 375k km power line
**This study**	**2008**–**2018**	**Weather, soil**	**Soil temperature**	**OMS**	**Extratropical and thunderstorms**	**3.3M customers, 375k km power line**

### 1.3 Objective and contributions

This study has two objectives. The first objective is to determine the effect of soil frost on wind-induced interruptions to assess the reliability of the electricity distribution system. The second objective is to define the best-correlating variables for soil frost and wind speed. The analysis is carried out with the programming language R and MS Excel Data Analysis Toolpack. The effects are analyzed for five case areas in Finland.

The main contributions of this paper are as follows:

1. The effect of soil frost and wind speed on the distribution network interruption frequency is determined.
2. The correlation of multiple wind and soil frost variables is investigated, and the optimal power relation of wind speed and interruption frequency is defined.

A further outcome of the present study is that the analyses are based on an extensive amount of statistical data on power supply interruptions from a long period.

### 1.4 Structure of the paper

Section 2 provides the background for electricity supply interruptions. Section 3 describes the methodology used in the analyses. Section 4 shows the results of the regression analyses. Section 5 discusses the results of the paper, and finally, Section 6 summarizes the findings of the paper.

## 2 Background

**Fig. 1:**
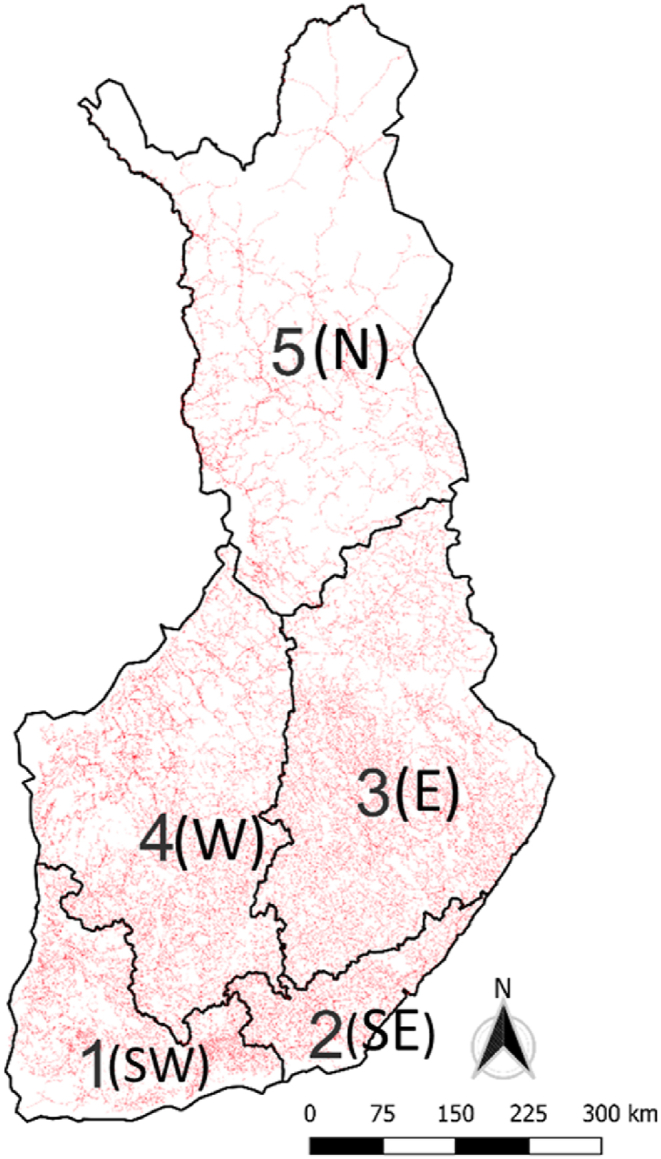
Areas of Finland based on the interruption dataset. Red lines represent the medium-voltage overhead line network of Finland.

The analysis is based on extensive interruption statistics from the years 2008–2018 gathered from Finnish electricity distribution system operators (DSOs). The data are divided into five areas shown in Fig. 1. The areas are numbered from 1 to 5, and the letters in brackets refer to the geographical location of the area (1(SW): southwestern Finland, 2(SE): southeastern Finland, 3(E): eastern Finland, 4(W): western Finland, and 5(N): northern Finland). Hereafter, we refer to these areas by their number and letter code. The regional division is based on the requirement of anonymization of the local DSOs and their customers. The condition for data sharing for research purposes requires a minimum of six DSOs operating in each area. The data contain all types of fault interruptions, and the causes of interruptions are divided into several groups, such as interruptions caused by wind, snow loads, or lightning. Non-meteorological causes like animals are also listed. This study focuses solely on interruptions caused by wind. The data cover over 95 % of the interruption events in the Finnish electricity distribution networks in a typical year. The exception is the year 2015, when a change was made in the method of data collection, and thus, the data coverage was low. In this context, the term interruption refers to a situation where one or more customers experience an interruption of power supply that lasts at least 3 min.

### 2.1 Data

The methodology developed in this paper is based on four datasets: an interruption dataset gathered from Finnish DSOs, the DSOs’ technical statistics dataset obtained from the Finnish Energy Authority (EA), wind speed data (average and wind gust speed measurements) from the Finnish Meteorological Institute (FMI), and soil temperature data (ERA5-Land reanalysis) from the European Centre for Medium-Range Weather Forecasts (ECMWF). All data, apart from the interruption dataset, are open data. The interruption dataset is not publicly available, and it is provided with the location data anonymized to five different areas containing a minimum of six DSOs to ensure anonymity. All the data were thus aggregated to the same level as the interruption dataset. The data cover the years 2008–2018, for a total of 4018 days.

#### 2.1.1 Interruption and network data

The interruption dataset includes information of the location (area), type, cause, time, number of customers affected, and length of the interruption for every interruption reported by the Finnish DSOs. The dataset was filtered to include only the wind-induced interruptions in the overhead line network, because they are in the focus of this study. The data were aggregated to a temporal resolution of one day. The technical statistics dataset from the Finnish Energy Authority was used to obtain the total medium-voltage overhead line lengths of the different areas for each year under study. The interruption rate (number of interruptions per day per kilometer) of the medium-voltage overhead line (OHL) network was then calculated based on these datasets and used as the target variable in the study.

#### 2.1.2 Environmental data

In the analysis, a total of nine variables were used: the number of wind-induced interruptions, the length of overhead lines, three soil temperature variables, and four wind speed variables. The soil temperature is determined at three different depths, whereas the different wind speed variables result from different ways to aggregate the data across measurement stations and time, as detailed in Section 3.2.

Hourly wind speed (average wind speed and wind gust speed) measurements were obtained from the open database of the Finnish Meteorological Institute [50]. The wind speed dataset included measured wind speeds from 450 stations. The data were aggregated to daily values.

Soil temperatures in three different depths (0–7 cm, 7–28 cm, and 28–100 cm) were obtained from the ERA5-Land meteorological reanalysis data produced by the ECMWF. Meteorological reanalysis datasets aim to represent past weather and climate conditions with complete data coverage [51]). Reanalysis datasets are produced by combining meteorological observations with forecasts of numerical weather prediction models. ERA5-Land has an hourly temporal resolution and a spatial resolution of 0.08 × 0.08°, which is approximately 9 × 9 km at the equator. To our knowledge, only a very limited number of studies have discussed the accuracy of ERA5-Land soil temperatures, and in Finland, such a comparison has only been made to the predecessor of ERA5-Land, ERA Interim, which had a much lower spatial and temporal resolution than ERA5-Land. In the master's thesis of Rimali [52], the resolution of the soil temperature data was higher compared with the very coarse resolution of ERA Interim. Previous studies have used the soil temperature of ERA5-Land Lembrechts et al. [53], and thus, the decision was to use ERA5-Land also in this study. The data are more easily accessible than the national, not openly available soil frost observation data of the Finnish Environment Institute.

### 2.2 Background analyses

Interruptions are divided into five geographical areas defined in the study (Fig. 1). The characteristics of the areas are shown in Table 2. The areas roughly correspond to the network areas of the DSOs; however, in the case of large DSOs, the network areas are split into smaller sections that are located in different areas. The data of these DSOs are divided into areas in proportion to the overhead line length having an accurate coordinate-based location based on the open data provided by the National Land Survey of Finland (NLS, 2019). The network characteristics contain medium-voltage network lines, which are mainly 20 kV lines.

**Table 2:** Characteristics of five geographical areas in Finland. In the table, the term weatherproof is used for overhead line sections for which the average tree height in the proximity is less than the height of the powerlines.^1)^ (EA, 2019),^2)^ (LUKE 2019)^,3^ (FMI, 2022).

	Area 1 (SW)	Area 2 (SE)	Area 3 (E)	Area 4 (W)	Area 5 (N)	Total
Network length, total (km)^1)^	30 823	11 431	41 112	49 014	20 526	152 906
Network length, overhead line (OHL) (km)^1)^	12 864	8368	33 839	28 838	14 358	98 267
Network length, underground cables (km)^1)^	17 959	3062	7272	20 177	6168	48 470
Weatherproof OHL (km)^1,2)^	5415	2345	3507	7770	2338	21 375
Proportion of weatherproof OHL (%)^1,2)^	42 %	28 %	10 %	27 %	16 %	22 %
Number of customers (millions)^1)^	1.19	0.34	0.68	1.05	0.39	3.66
OHL length per customer (m/customer.)^1)^	11	24	50	27	37	27
Average height of trees (m)^2)^	15.7	13.9	14.4	15.6	9.6	13.4
Average temperature in a year (°C)^3)^	5.8	5.3	4.2	3.7	1.9	4.2
Days below 0 °C in a year (1/a)^3^	80	95	108	108	147	538
Average peak wind gust on a day (m/s)^3^	11.8	11.1	8.9	10.4	10.1	10.5
Number of meteorological stations	123	40	96	104	87	450

The areas differ significantly from each other; for example, Area 5(N) is located in northern Finland, which is sparsely populated. The network structures are also different between areas, and the environmental locations of the lines vary. In the southern and western parts of the country, the remaining overhead lines are more often located in fields, whereas elsewhere in the country they are mainly built in forests. For instance, the largest proportion of weatherproof overhead lines is found in Area 1(SW) located in southwestern Finland. This makes a significant difference in the failure probability of a line. Furthermore, in the southern parts of the country, a large proportion of overhead lines have been replaced by underground cables over the past ten years. Thus, for a fair comparison, the annual interruption statistics are weighted by the annual overhead line lengths to ensure that the decreasing overhead line length because of the increasing underground cable length is correctly taken into consideration. The term weatherproof is used for overhead line sections for which the average tree height in the proximity is less than the height of the powerlines.

**Fig. 2:**
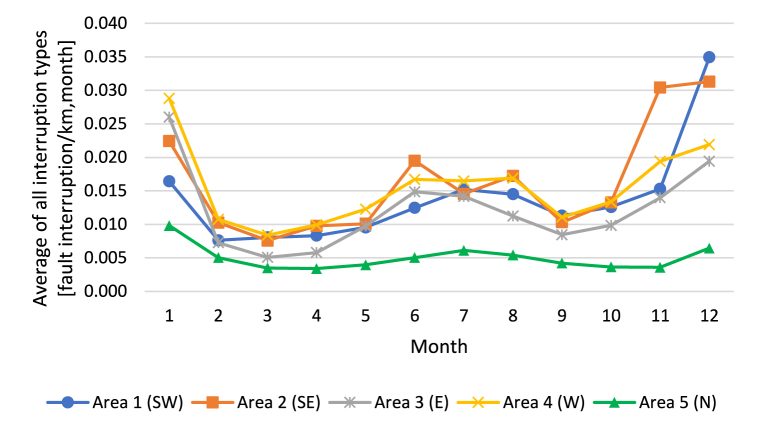
Average number of all monthly interruptions per overhead line length experienced by electricity end users.

**Fig. 3:**
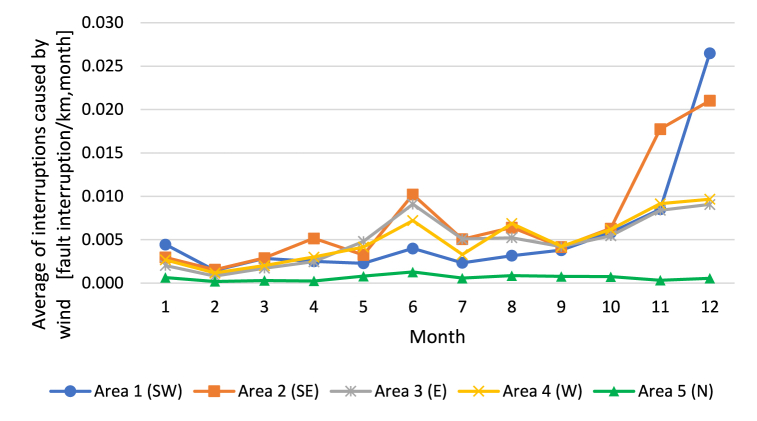
Average number of monthly wind-induced interruptions per overhead line kilometer experienced by electricity end users.

The highest wind gusts are found in Area 1(SW), where the daily average peak gust is 11.8 m/s. The wind gusts are lowest in Area 3(E), being 25 % lower than in Area 1(SW). Moreover, there is significant seasonal variation in interruptions of supply in the electricity distribution systems. This is illustrated in Fig. 2, Fig. 3, which show the average numbers of interruptions caused by all sources of faults and interruptions caused by wind. The interruption frequencies are presented in proportion to the overhead line length.

It can be observed from Fig. 2, Fig. 3 that the occurrences of interruption are close to each other in Areas 1–4. The same result is seen with the statistics of interruptions caused by all sources of faults (Fig. 2) and interruptions caused by wind (Fig. 3). The figures indicate that interruptions occur more often in autumn and mid-winter. In mid-summer, the number of interruptions is also higher than in spring and at the beginning of autumn. In the Finnish conditions, wind-induced interruptions are emphasized in autumn and mid-summer. The hypothesis is that soil frost reduces the number of wind-induced interruptions in winter and spring; however, the number of snow-induced interruptions increases when snow loads increase the stress and number of interruptions in the overhead line network.

Ten storms that have caused most electricity interruptions in Finland between 2008 and 2018 are listed in Table 3. It can be seen that these storms caused 28 % of all the wind-induced interruptions, whereas wind gusts of the other storms caused the rest 72 % of the wind-induced interruptions.

**Table 3:** Power supply interruptions in 2008–2018 caused by ten strongest windstorms.

Number of interruptions caused by wind	Proportion (%)	Date	Name of windstorm
5911	7.2 %	26 Dec. 2011	Tapani
3253	4.0 %	13 Dec. 2013	Seija
3167	3.9 %	17 Nov. 2013	Eino
2932	3.6 %	27 Dec. 2011	Hannu
1491	1.8 %	30 Nov. 2012	Antti
1449	1.8 %	27 Aug. 2016	Rauli
1403	1.7 %	23 Nov. 2008	not named
1269	1.6 %	2 Oct. 2015	Valio
926	1.1 %	1 Dec. 2013	Oskari
920	1.1 %	4 Jun. 2009	Toivo
**58** **884**	**72.2 %**	**-**	**Others**

## 3 Methodology

In this section, the methodology to analyze the effect of wind speed and soil frost on faults in the electricity distribution network is presented. There are numerous methods available for the analysis; however, the study focuses on the linear regression model. It was selected as the methodology to estimate the effect of wind speed and soil frost on power system interruptions. The reason behind selecting the linear regression model is that it is well known, and it provides a transparent analysis to evaluate results based on limited amount of input variables [54]. Thus, the results can be easily incorporated in power system planning and asset management. The other models, such as different decision trees or machine learning models, may yield a higher correlation and slightly better estimates; however, they may not necessarily enhance the usability of the results.

### 3.1 Statistical analyses

The frequency of interruptions, i.e., the number of interruptions per day per km of overhead line, was the object of primary interest. To this end, the daily numbers of interruptions were divided by the length of the overhead line. The data were analyzed separately for each of the five areas. Pearson's product-moment correlation coefficient and the linear model [55] were used to analyze the data.

The marginal effects of different variables were calculated as follows. First, consider a linear univariate model for interruption frequency

(1) $$y_{i}=\alpha_{i}+\beta x_{i}+\varepsilon_{i}\;\text{.}$$

In such a model, one unit of x changes y by the amount coefficient β in area *i*, whereas α is constant, and ε is an error term. For the linear model, the term β is the effect size. The variable x can be wind speed, the power of wind speed, or soil frost. The confidence interval (CI) of the coefficient β determined in the study was calculated as

(2) $$CI=\left[\beta_{low},\beta_{high}\right]\text{,}$$

where $\left[\beta_{low},\beta_{high}\right]$ is the usual confidence interval of β [55]. In these analyses, the response variable is the frequency of interruptions. It represents the average number of interruptions in each area per day per power line kilometer.

In this paper, the significance of the results is considered by using the P-value. In addition, to avoid the peril of P-value hacking, the P-values were multiple testing corrected by the false discovery rate (FDR) method presented in Ref. [56]. The FDR method provides another approach to confirm the validity of the results, while being less overly conservative than the Bonferroni correction.

Further, the paper considers a multivariate model with an interaction term [57]. The model assesses the combined effect of wind speed and soil frost on the power network interruption frequency

(3) $$y_{i}=\alpha +\beta_{1}x_{1i}+\beta_{2}{x_{2i}x}_{1i}+\varepsilon_{i}\text{,}$$

where one unit of x_1_ affects y by the amount $\beta_{1}$ in area *i,* multiplication of the binary variable x_2_, and the variable x_1_ affects y by the amount $\beta_{2}$ in area *i*. In the model, x_1_ is the wind speed or its power, $\beta_{1}$ is the effect of wind speed, x_2_ is the binary variable of soil frost, and $\beta_{2}$ is the effect of soil frost. The definitions previously considered for the confidence intervals of the univariate model related to the determination of the coefficients are valid for the multivariate linear models.

### 3.2 Effect of wind speed

In the analyses, two primary wind speed variables were used to determine the best correlation between wind speed and occurrence of interruptions. The speed variables are the 10 min average wind speed (WS) and the maximum of the 3 s wind speed for a 10 min measurement period (WG) for each measurement station. From the 10 min values, the maximum values were determined for each hour. In the analyses, daily average and maximum values of the wind speed variables were used. From these values, regional values were determined for the wind speed variables.

To determine the best correlation between wind speed and the number of wind-induced electricity interruptions, we considered four ways to aggregate the data. Both the WS and the WG were taken into consideration by calculating the daily average or maximum values for the five regions under study based on measurements obtained from several stations. In the analysis, also the use of cubes of the variables was considered because the power delivered by wind is proportional to the third power of wind speed. This can be derived from the kinetic energy of a moving mass:

(4) $$E_{k}=\frac{1}{2}mv^{2}\text{,}$$

where v is the speed. To solve the mass m, consider a surface perpendicular to the flow (e.g., the canopy of a tree). The mass flux through this surface is

(5) $$\dot{m}=\rho Av\text{,}$$

where ρ is the density of the air and A is the area of the surface. Thus, the mass equals

(6) $$m=\Delta t\dot{m}\text{,}$$

where Δt is the time interval. The power is given by

(7) $$P=\frac{E_{k}}{\Delta t}=\frac{\frac{1}{2}\Delta t\dot{m}v^{2}}{\Delta t}=\frac{1}{2}\rho Av^{3}\propto v^{3}\text{.}$$

However, the literature indicates that the relation between wind speed and wind damage can be higher than the third power [32]. Thus, we examined the correlation of the wind speed and the number of power interruptions with powers between 1 and 20. The aggregations of wind speed variables used in the analysis are described in Table 4.

**Table 4:** Aggregation of different wind speed measures in the analysis.

Option	Definition	Variable	Aggregation (station level)	Aggregation (time level)
1	*WS*_max,max_	10-min wind speed	Maximum	Daily maximum
2	*WS*_ave,max_	10-min wind speed	Average	Daily maximum
3	*WG*_max,max_	Wind gust	Maximum	Daily maximum
4	*WG*_ave,max_	Wind gust	Average	Daily maximum

The definitions and explanations of the wind speed under consideration were determined by the following formulations:

- *WS*_max,max_, the daily maximum of the hourly maximum taken over the maximum 10 min wind speeds at the measurement stations in the area
- *WS*_ave,max_, the daily maximum of the hourly average taken over the maximum 10 min wind speeds at the measurement stations in the area
- *WG*_max,max_, the daily maximum taken over the maximum wind gusts at the measurement stations in the area
- *WG*_ave,max_, the daily maximum of the hourly average taken over the maximum wind gusts at the measurement stations in the area

(8) $${{WS}_{\max\_\max}}^{a}={\left(\max_{t}\;\max_{i}{WS}_{i,t}\right)}^{a},i=1\ldots N,t=1\ldots T,a=1...20$$

(9) $${{WS}_{\text{ave}\_\max}}^{a}={\left(\max_{t}\frac{\sum_{i=1}^{N}{WS}_{i,t}\;}{N}\right)}^{a},t=1\ldots T,a=1...20$$

(10) $${{WG}_{\max\_\max}}^{a}={\left(\max_{t}\;\max_{i}{WG}_{i,t}\right)}^{a},i=1\ldots N,t=1\ldots T,a=1...20$$

(11) $${{WG}_{\text{ave}\_\max}}^{a}={\left(\max_{t}\frac{\sum_{i=1}^{N}{WG}_{i,t}\;}{N}\right)}^{a},t=1\ldots T,a=1...20$$

where *a* is the power of wind speed, *i* is the group of measurement stations within the area under consideration, *N* is the number of measurement stations, *t* is the group of time periods within a day, and *T* is the number of time periods within a day.

**Fig. 4:**
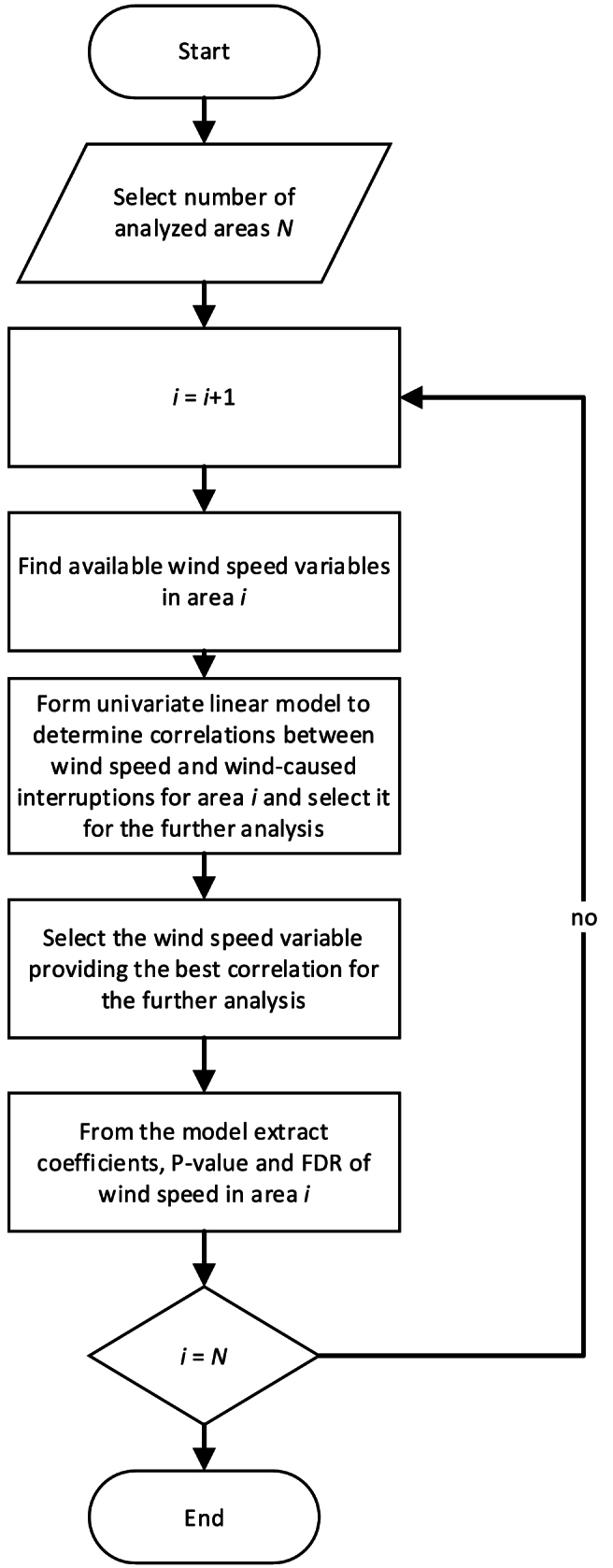
Methodology to determine the statistical dependence between wind speed and occurrence of interruptions in electricity distribution.

The methodology to determine the dependence of wind speed and the number of fault interruptions is shown in Fig. 4.

### 3.3 Method to analyze the effects of soil frost

**Fig. 5:**
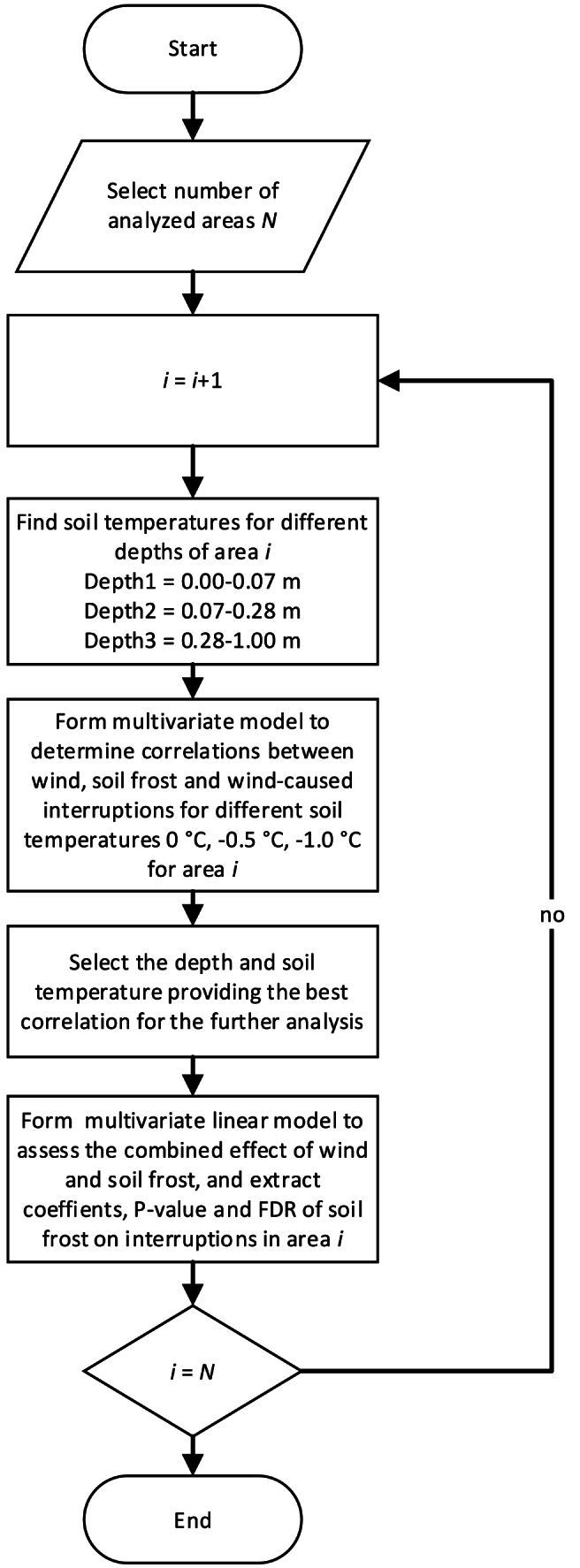
Methodology to determine the effect of soil frost on the reduction in wind-induced interruptions.

The effect of soil frost is assessed with the selected wind speed aggregation determined in the previous analysis concerning wind speed. In this paper, the analysis is carried out with soil temperature information from the ERA5-Land reanalysis, because the information is openly available and the actual soil frost is only sparsely measured. The soil temperature was compared from each depth (0–7 cm, 7–28 cm, and 28–100 cm) with three cut-off values of the soil temperature, 0 °C, −0.5 °C, and −1.0 °C, respectively. For simplicity, it was assumed that soil frost is present if the soil temperature is below the set temperature cut-off value. Pearson's product moment correlation coefficients were calculated for the multivariate linear regression model where the wind is one explanatory factor for the wind-induced interruptions and soil frost-based variable is another factor. The effect of soil frost is determined as the multiplication of wind speed aggregation and binary frost variable. Soil frost correlation is assessed for each soil temperature cut-off value and depth. The methodology to select the best-correlating soil frost depth and temperature is presented in Fig. 5.

## 4 Results

The results of the study are based on the methodology presented in the previous section.

### 4.1 Effects of wind on the number of faults in the electricity distribution network

As a result of the analysis, correlation coefficients were determined for the wind speed variables used in the study. The correlation of the wind speed and fault interruptions was calculated with powers between 1 and 20. The results of powers 1, 3, and 10 are presented in Table 5 and the powers between 1 and 20 in Appendix Table A1.

**Table 5:** Pearson's product moment correlation coefficients of the number of wind-induced interruptions and different definitions of wind speed. The highest correlations are in boldface.

	Area 1 (SW)	Area 2 (SE)	Area 3 (E)	Area 4 (W)	Area 5 (N)	Average
*WS*_max,max_	0.16	0.23	0.31	0.30	0.18	0.24
*WS*_ave,max_	0.18	0.24	0.32	0.33	0.27	0.27
*WG*_max,max_	0.17	0.25	0.33	0.31	0.22	0.26
*WG*_ave,max_	0.19	0.25	0.33	0.34	0.28	0.28
*WS*_max,max_^3^	0.23	0.36	0.46	0.43	0.11	0.32
*WS*_ave,max_^3^	0.29	0.36	0.49	0.49	0.36	0.40
*WG*_max,max_^3^	0.25	0.38	0.47	0.45	0.26	0.36
*WG*_ave,max_^3^	0.32	0.39	0.51	0.51	0.37	0.43
*WS*_max,max_^10^	0.36	**0.63**	0.7	0.63	<0.01	0.58
*WS*_ave,max_^10^	0.72	0.39	0.69	0.75	0.4	0.59
*WG*_max,max_^10^	0.39	0.6	0.36	0.63	0.07	0.41
***WG***_**ave,max**_^**10**^	**0.8**	0.61	**0.77**	**0.77**	**0.40**	**0.67**

Based on the results (Table 5 and Appendix Table A1), *WG*_ave,max_^10^ is the wind speed variable providing the best correlation for the majority of the areas considered in the study. The correlation coefficients of the tenth power are significantly higher compared with the smaller first and third powers. Moreover, in general, it can be stated that the hourly average wind speed (both wind gusts and 10 min wind speed) over the measurement stations in the area provides a higher correlation compared with the maximum wind speed over the measurement stations in the area. The reason for this is that the larger the area influenced by the windstorm is, the wider the effects of the storm are. A closer consideration of the best correlations reveals that in one of the areas, Area 1(SW), the best correlation is found with *WG*_ave,max_^20^.

The effect of wind gusts on the occurrence of interruptions is presented in Table 6, which shows the results of a univariate regression model fitted for the wind speed. It shows the coefficient of wind speed, its confidence intervals, P-value, FDR value, and the modeled average interruption frequency for the areas under study. The interruption frequency is determined with the model, and the wind gust distribution from a period of 11 years.

**Table 6:** Effect of wind speed on the number of wind-induced interruptions. The effects were estimated from a regression model using the wind speed aggregation *WG*_ave,max_^10^. CI-low = 2.5 % confidence interval, CI-high = 97.5 % confidence interval.

	Wind speed coefficient β	CI-low	CI-low	P-value	FDR	Modeled interruption frequency (day^-1^1000km^−1^)
Area 1 (SW)	2.8E-13	2.7E-13	2.9E-13	<0.001	<0.001	0.32
Area 2 (SE)	3.1E-13	3.0E-13	3.3E-13	<0.001	<0.001	0.23
Area 3 (E)	1.7E-12	1.6E-12	1.7E-12	<0.001	<0.001	0.12
Area 4 (W)	3.5E-13	3.4E-13	3.6E-13	<0.001	<0.001	0.12
Area 5 (N)	3.0E-12	2.7E-12	3.2E-12	<0.001	<0.001	0.01

The P-value and FDR determined in the study indicate that the results are significant even though the effect sizes vary between the areas. Based on the analysis, the coefficients of wind are between 2.8E-13 and 3.0E-12 on average, depending on the area under consideration. Considering the interruption frequency, the numbers of interruptions vary between 0.01 and 0.32 interruptions per day per 1000 km of overhead line. It can be observed that the farther south the area is, the more interruptions are caused by wind. This is reasonable because the trees are taller (Table 1) and soil frost is less common in the south, and thus, it has a lower effect in the south. It can be noticed that the modeled interruption frequency of Area 1(SW) is higher compared with the other areas. This can partly be explained by one major storm having the strongest effect in Area 1(SW). The statistics also show that one major storm in December 2011 (named Tapani in Finland and cyclone Dagmar in the other Nordic countries) caused 25 % of all wind-induced interruptions.

**Fig. 6:**
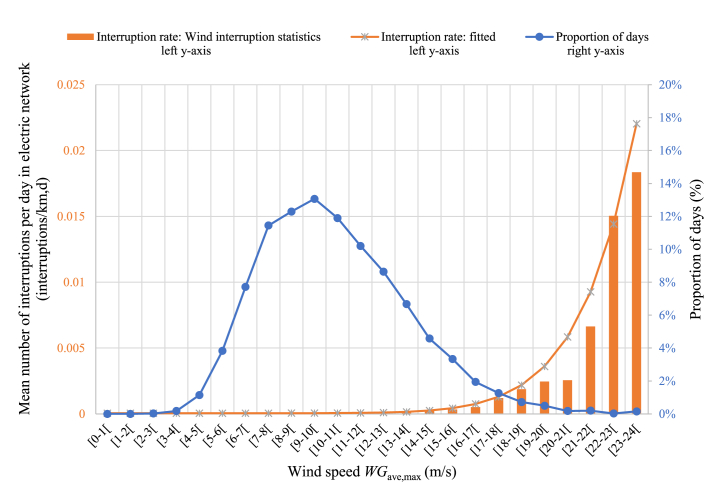
Effect of wind speed on daily interruptions in Area 4(W). In the analysis, the wind speed *WG*_ave,max_ is the daily maximum of the hourly averages of the maximum wind gusts at the measurement stations in the area. The data are from the years 2008–2018, consisting of 4018 days and 22 881 interruptions.

Fig. 6 presents the distribution of wind gusts occurring in Area 4(W), the numbers of interruptions caused by different wind speeds, and the fitted number of interruptions based on the selected tenth power of *WG*_ave,max_. The data are from a period of 11 years between 2008 and 2018.

**Fig. A.1:**
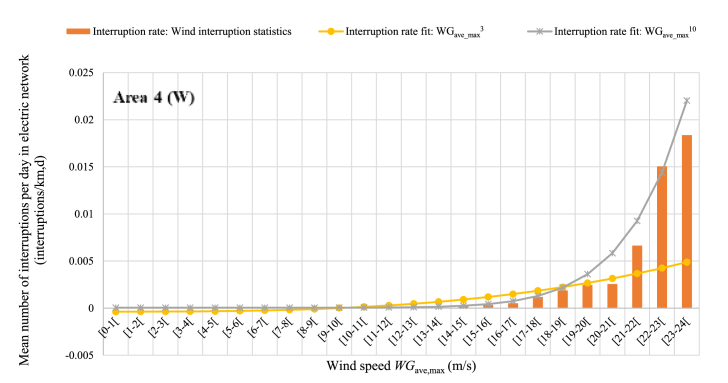
Interruptions caused by wind in Area 4(W). The bars show the mean number of interruptions, and the line diagrams illustrate two different fits for the daily mean number of interruptions with the wind speed *WG*_ave,max_, which is the daily maximum of average hourly measured maximum wind gusts. The fits are for the third and tenth powers of *WG*_ave,max_. The data are from the years 2008–2018, consisting of 4018 days and 22 881 interruptions.

The figure shows that the wind speed *WG*_ave,max_ has almost a normal distribution, but the interruptions caused by wind occur with high wind speeds so that the probability of an interruption increases exponentially when the wind speed increases. Thus, a small number of highly windy days cause most of the wind-induced interruptions. In Area 4(W) this means, for instance, that 64 % of all wind-induced fault interruptions occur on days when the maximum wind speed is 15 m/s or higher, such days accounting for 8 % of all days. On average, for all the areas these percentages are 68 % and 10 % for the wind speed of 15 m/s or higher. The same percentage values, on average, with the wind speed of 10 m/s or higher are 92 % and 51 %, respectively. The figure also shows that the model fits the dataset. Fig. A1 in Appendix 1 shows a comparison of the two fits *WG*_ave,max_^3^ and *WG*_ave,max_^10^, and the conclusion is that the fit with the tenth power is better.

### 4.2 Combined effect of soil frost and wind speed with multivariate linear model

The effect of soil frost is assessed in this analysis by multivariate linear regression models. The soil frost analyses are based on the following numbers of days and fault interruptions with and without soil frost presented in Table 7.

**Table 7:** Numbers of days and fault interruptions in all the areas under consideration with and without soil frost during a 11-year period.

	No soil frost, number of days	Soil frost, number of days	No soil frost, number of fault interruptions	Soil frost, number of fault interruptions
Area 1 (SW)	3457	561	21 979	1273
Area 2 (SE)	3106	912	8649	413
Area 3 (E)	2718	1300	21 784	3628
Area 4 (W)	2924	1094	20 984	1897
Area 5 (N)	2154	1864	685	313

#### 4.2.1 Determination of the best-correlating soil frost depth and temperature

First, we determine the best combination of soil temperature and soil depth to describe soil frost by the methodology shown in Section 3.3. This provides the correlations for each depth and cut-off temperature for each of the geographical areas. In the model, the wind speed aggregation *WG*_ave_max_^10^, which was determined in the previous analysis concerning wind speed, is used to estimate the failure of the power network. In addition, the effect of soil frost is estimated by using the binary variable of soil frost, which is multiplied by the wind speed aggregation *WG*_ave_max_^10^. The calculated correlation coefficients between soil frost and wind-induced interruptions based on the methodology applied in the study are given in Table 8.

**Table 8:** Correlation of the number of wind-induced interruptions and different definitions of frost. The values are Pearson's product moment correlation coefficients. The depth levels from 1 to 3 correspond to the depths of 0–7 cm, 7–28 cm, and 28–100 cm, respectively. The highest correlations are in boldface.

	Area 1 (SW)	Area 2 (SE)	Area 3 (E)	Area 4 (W)	Area 5 (N)	Average
Depth 0–7 cm, cut-off 0 °C	0.806	0.621	0.804	**0.839**	0.535	0.721
Depth 0–7 cm, cut-off −0.5 °C	0.803	0.619	0.786	0.818	0.435	0.692
Depth 0–7 cm, cut-off −1.0 °C	0.803	0.615	0.794	0.770	0.410	0.678
Depth 7–28 cm, cut-off 0 °C	**0.810**	0.615	0.807	0.827	**0.543**	0.720
Depth 7–28 cm, cut-off −0.5 °C	0.807	**0.633**	0.813	0.830	0.541	**0.725**
Depth 7–28 cm, cut-off −1.0 °C	0.803	0.627	0.803	0.772	0.459	0.693
Depth 28–100 cm, cut-off 0 °C	0.805	0.625	**0.824**	0.768	0.492	0.703
Depth 28–100 cm, cut-off −0.5 °C	0.802	0.616	0.772	0.768	0.435	0.679
Depth 28–100 cm, cut-off −1.0 °C	0.802	0.615	0.771	0.768	0.406	0.672

The analysis shows that soil frost improves the correlation of the model in all the depths and cut-off temperatures compared with the univariate only wind speed aggregated regression model (Table 9). The results show that the best correlation in Areas 1–5 is achieved with different depths and cut-off temperatures. On average, the best correlation is provided by the depths of 7–28 cm with the cut-off of −0.5 °C, and thus, this correlation was chosen to be used in the further regression analysis.

**Table 9:** Correlations of the univariate linear regression model with the number of wind-induced interruptions and the wind speed aggregation *WG*_ave,max_^10^ and the multivariate linear regression model with the number of wind-induced interruptions, the wind speed aggregation *WG*_ave,max_^10^, and soil frost. The values are Pearson's product moment correlation coefficients.

	Area 1(SW)	Area 2(SE)	Area 3(E)	Area 4(W)	Area 5(N)
Correlation R: Univariate model *WG*_ave,max_^10^	0.8	0.61	0.77	0.77	0.40
Correlation R: Multivariate model *WG*_ave,max_^10^ + soil frost depth 7–28 cm, cut-off −0.5 °C	0.81	0.63	0.81	0.83	0.54

It can be observed that the correlation coefficients increased in most of the areas. The lowest increase in correlation is found in Area 1(SW) and Area 2(SE), where the increase of the correlation is below 0.02. The lowest increase in correlation in Areas 1 and 2 can partly be explained by the lowest number of days with soil frost. In addition, the quality of data affects the results.

#### 4.2.2 Assessment of wind speed and soil frost coefficients

The analysis now determines the effect of wind and soil frost on the interruption frequency of an overhead electricity distribution line. The results of the effect of wind speed are given in Table 10, and the results of the combined effect of soil frost and wind speed in Table 11. Like in the previous analysis for the wind-induced interruptions, the tables show the coefficients, confidence intervals, P-value, FDR value, and average interruption frequency for the areas under study. The interruption frequency is determined with the model and distribution of wind gusts from the 11-year time period.

**Table 10:** Effect of wind speed on the number of wind-induced interruptions in the multivariate model. The effects were estimated from a linear regression model using the wind speed aggregation *WG*_ave,max_^10^. CI-low = 2.5 % confidence interval, CI-high = 97.5 % confidence interval.

	Wind speed coefficient $\beta_{1}$	CI-low	CI-high	P-value	FDR	Average interruption frequency (day^-1^1000km^−1^)
Area 1(SW)	2.8E-13	2.8E-13	2.9E-13	<0.001	<0.001	0.32
Area 2(SE)	3.3E-13	3.2E-13	3.5E-13	<0.001	<0.001	0.24
Area 3(E)	2.0E-12	1.9E-12	2.0E-12	<0.001	<0.001	0.15
Area 4(W)	4.2E-13	4.1E-13	4.2E-13	<0.001	<0.001	0.15
Area 5(N)	8.4E-12	7.9E-12	8.8E-12	<0.001	<0.001	0.03

**Table 11:** Average effect of soil frost on the number of wind-induced interruptions during soil frost in the multivariate model with the wind speed aggregation *WG*_ave,max_^10^. The effects are estimated from a linear regression model. CI-low = 2.5 % confidence interval, CI-high = 97.5 % confidence interval.

	Soil frost coefficient $\beta_{2}$	CI-low (day^-1^1000km^−1^)	CI- high (day^-1^1000km^−1^)	P-value	FDR	Average effect on interruption frequency (day^-1^1000km^−1^)
Area 1(SW)	−2.0E-13	−2.0E-13	−1.3E-13	<0.001	<0.001	−0.16
Area 2(SE)	−2.8E-13	−3.2E-13	−2.3E-13	<0.001	<0.001	−0.14
Area 3(E)	−1.3E-12	−1.4E-12	−1.2E-12	<0.001	<0.001	−0.08
Area 4(W)	−3.6E-13	−3.8E-13	−3.4E-13	<0.001	<0.001	−0.11
Area 5(N)	−6.7E-12	−7.1E-12	−6.2E-12	<0.001	<0.001	−0.03

Table 10 shows that the coefficients and the interruption frequencies are close to the results of the univariate model for the aggregated wind speed *WG*_ave,max_^10^. The results are significant in all areas.

The analysis shows that the effect of soil frost on the wind-induced interruptions is negative everywhere, providing coefficients between −6.7E-12 and -2.0E-13 and effects of soil frost on the interruption frequency between −0.16 and −0.03 interruptions per day per 1000 km of overhead line. This means that soil frost reduces the number of wind-induced power system interruptions. The results are statistically significant in all areas. It is noteworthy that the reduction in interruptions is present only for the days when the soil is frozen, i.e., in the model the soil temperature is below −0.5 °C at the depth of 7–28 cm. Thus, this effect is not present in the summertime.

**Fig. 7:**
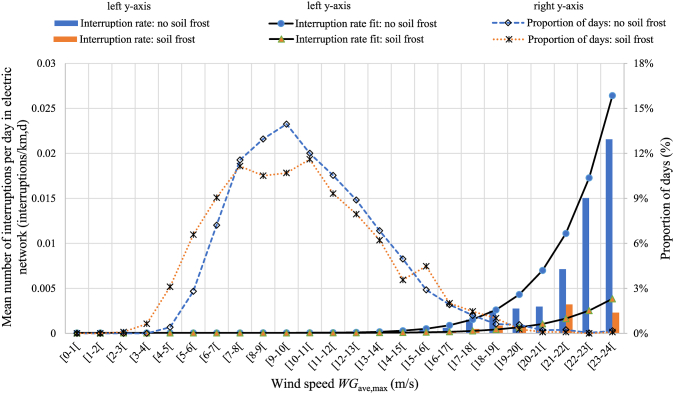
Interruptions caused by wind in Area 4(W). The bars show the mean number of interruptions, and the line diagrams depict the proportion of days with the wind speed *WG*_ave,max_, which is the daily maximum of average hourly measured maximum wind gusts. The data contain 2924 (73 %) days without soil frost and 1094 (27 %) days with soil frost. The total number of interruptions in the electricity distribution network was 20 984 (92 %) without soil frost and 1897 (8 %) with soil frost.

Fig. 7 illustrates both the actual and fitted interruption rates in Area 4(W) caused by wind in two cases: first, there is no soil frost, and second, soil frost is present. In addition, the proportions of days sorted by wind speed (*WG*_ave,max_) are presented. Appendix 1 provides figures for the other geographical areas with the actual interruption rates and wind speed distributions.

It can be observed that the fitted interruption rates match up with the actual interruption rates. However, with the high wind speeds the fits slightly overestimate the number of interruptions. In addition, the figure shows that soil frost considerably reduces the number of interruptions. The reduction in the number of faults compared with the wind-induced interruptions without soil frost is shown in Table 12.

**Table 12:** Reduction in the proportion of wind-induced interruptions when soil frost is present.

	Coefficient $\beta_{2}$ of soil frost interaction term	Wind speed coefficient $\beta_{1}$	Effect of soil frost on wind-induced interruptions (%)	Days with soil frost, depth 7–28 cm cut-off −0.5 °C (avg days per year)	Days with soil frost, depth 7–28 cm cut-off −0.5 °C (%)
Area 1(SW)	−2.0E-13	2.8E-13	−71 %	51	14 %
Area 2(SE)	−2.8E-13	3.3E-13	−83 %	83	23 %
Area 3(E)	−1.3E-12	2.0E-12	−66 %	118	32 %
Area 4(W)	−3.6E-13	4.2E-13	−86 %	99	27 %
Area 5(N)	−6.7E-12	8.4E-12	−79 %	1169	46 %

The analysis shows that the reduction in percent is between 66 % and 86 %, which is a relatively significant effect. However, it is pointed out that soil frost is present only for a short period in a year. For instance, in Area 5(N), the proportion of days with soil frost is 46 %, meaning that for more than half a year soil frost does not reduce the number of wind-induced interruptions. In Areas 1–4 the proportion of soil frost days was between 14 % and 32 % in the years 2008–2018.

## 5 Discussion

Wind causes a significant proportion of supply interruptions in an electricity distribution system that is based on overhead lines. The analyses presented in this paper rely on extensive interruption statistics of the Finnish electricity distribution system for a period of 11 years.

Our analysis reveals that the best correlation between the number of supply interruptions and wind speed is found with the tenth power of the wind gust (3 s average wind speed), with a strong correlation in most areas (on average 0.67). This corresponds to a previous study of the effect of wind speed on forest damage, where the best power relation was found to be approximately 10 [32]. While forest damage and electricity grid interruptions have characteristics of their own, a similar result was expected, because the main cause of damage in Finland or other densely forested countries, i.e., falling of trees (windthrow), is the same. The number of interruptions was found to increase considerably when the wind speed rises above 20 m/s, whereas the statistics indicate that interruptions rarely occur when the daily maximum wind gusts are below 10 m/s.

Our analysis shows further that soil frost increases the correlation of the assessed multivariate model compared with the wind-based univariate model. On average, the correlation is 0.72 with soil frost. The effect of soil frost on the electricity distribution network interruptions caused by wind is significant, even 50 % fewer interruptions, if soil frost is present. However, the statistics show that the number of days when soil frost is present can be relatively low even in the Finnish conditions. Thus, the absolute effect on the number of interruptions can be low especially in the southern parts of Finland especially if the number of soil frost days decreases. Another noteworthy aspect related to soil frost is that in the analyses the geographical areas are relatively large and that mean values of frost are used. In reality, local soil frost conditions can vary considerably. This means that the correlation coefficients with frost could be higher if more accurate fault locations were known, and thus, a better estimate of soil frost condition could be used.

The warming climate leads to a shorter soil frost period and a reduction in the soil frost depth Venäläinen et al. [58], Gregow [29], Lehtonen et al. [21]. This also has an impact on the electricity distribution system, because soil frost will occur less often in areas where it has been common in the past. Thus, the study indicates that the number of supply interruptions caused by wind will increase in the future when the temperatures rise, especially in winter. Consequently, the number of wind-induced interruptions will increase if the amount of overhead lines remains at the same level. However, the ongoing replacement of overhead lines by underground cables reduces the OHL length.

The results show that the effect sizes of soil frost and wind on interruptions differ between the areas under study. This is an expected result because interruption frequencies are also affected by other environmental and technical factors not included in this study, such as tree heights near overhead lines, distinct vegetation management strategies, and the use of more fault-resilient overhead line technologies, such as coated overhead line conductors.

Although the amount of data on which the model is based is substantial, with the daily data of 11 years covering the whole area of Finland, the aggregation of data into five areas causes some loss of information. This is most apparent in Areas 1(SW) and 5(N) because the DSO has an electricity distribution network in both areas, whereas the interruptions of this DSO are aggregated only to Area 1(SW). This weakens the statistical significance of the results to some degree. Furthermore, a large proportion of the total number of interruptions are caused by a single large windstorm, having a significant effect on the statistics.

## 6 Conclusions

This study shows that soil frost has a significant impact on the number of wind-induced power system interruptions. In the previous studies, soil frost has not been incorporated in the interruption analyses. In this study, univariate and multivariate linear models were used to analyze the effect of wind speed and soil frost on the occurrence of interruptions in the electricity distribution system. The method was tested and verified with data gathered in Finland. The effects were analyzed for five areas in Finland. Four different wind and soil frost variables with different powers were considered, and the variables used in the regression analysis were selected based on Pearson's product-moment correlation coefficients. Coefficients of different variables were calculated based on linear models.

Based on the analysis, the interruption frequency of the wind-induced faults was between 0.03 and 0.33 interruptions/1000 km,d depending on the geographical area. The best correlation was found with the tenth power of daily maximum wind gusts. The reducing effect of soil frost on fault interruptions was found to be between −66 % and −86 %, while the best correlation on average was found to be with the soil temperature of below −0.5 °C in a depth of 7–28 cm. The result was statistically significant in all areas. The results of the analysis show that the presence of soil frost has a significant effect on the interruptions caused by wind. Nevertheless, the statistics show that the number of days when soil frost is present is relatively low even in the Finnish conditions.

The results of this study can be used to assess the impact of overhead lines on the customer interruption costs and long-term network planning. In the future, more accurate spatial data could provide better conditions for the analysis, and the methodology could be developed further by employing more advanced models, such as machine learning models. Furthermore, the improved quality of interruption datasets containing spatially accurate fault locations, additional environmental predictor variables, such as tree height, crown snow load, accurately measured indicators of soil frost, and wind direction along with network infrastructure variables, such as the amount of weatherproof overhead lines, could be added to the model. The dataset could also be used to study the combined effect of reliability-enhancing technologies, such as underground cabling. To investigate the effect of climate change and provide a more accurate analysis of the effects of soil frost on power systems, the analysis could be repeated by using wind and soil temperature, or additional parameter outputs from climate models.

## CRediT authorship contribution statement

**Juha Haakana:** Writing – review & editing, Writing – original draft, Visualization, Validation, Methodology, Formal analysis, Conceptualization. **Otto Räisänen:** Writing – review & editing, Writing – original draft, Visualization, Software, Methodology, Formal analysis. **Markku Karhunen:** Writing – original draft, Software, Methodology, Formal analysis. **Ilona Láng-Ritter:** Writing – original draft, Conceptualization. **Jukka Lassila:** Supervision.

## Data and code availability statement

The raw data required to reproduce the above findings cannot be shared at this time because the authors do not have permission to share the data.

## Declaration of competing interest

The authors declare that they have no known competing financial interests or personal relationships that could have appeared to influence the work reported in this paper.

## Figures and Tables

**Table A.1:** Pearson's product moment correlation coefficients of the number of wind-induced interruptions and different definitions of wind speed.

	Area 1 (SW)	Area 2 (SE)	Area 3 (E)	Area 4 (W)	Area 5 (N)	Average
*WS*_max,max_	0.16	0.23	0.31	0.3	0.18	0.24
*WS*_ave,max_	0.18	0.24	0.32	0.33	0.27	0.27
*WG*_max,max_	0.17	0.25	0.33	0.31	0.22	0.26
*WG*_ave,max_	0.19	0.25	0.33	0.34	0.28	0.28
*WS*_max,max_^3^	0.23	0.36	0.46	0.43	0.11	0.32
*WS*_ave,max_^3^	0.29	0.36	0.49	0.49	0.36	0.40
*WG*_max,max_^3^	0.25	0.38	0.47	0.45	0.26	0.36
*WG*_ave,max_^3^	0.32	0.39	0.51	0.51	0.37	0.43
*WS*_max,max_^4^	0.26	0.43	0.53	0.49	0.05	0.35
*WS*_ave,max_^4^	0.35	0.41	0.57	0.57	0.39	0.46
*WG*_max,max_^4^	0.29	0.45	0.52	0.51	0.25	0.40
*WG*_ave,max_^4^	0.39	0.46	0.59	0.59	0.41	0.49
*WS*_max,max_^5^	0.29	0.49	0.6	0.54	0.01	0.39
*WS*_ave,max_^5^	0.42	0.44	0.64	0.63	0.41	0.51
*WG*_max,max_^5^	0.32	0.5	0.55	0.56	0.23	0.43
*WG*_ave,max_^5^	0.47	0.52	0.66	0.66	0.4	0.54
*WS*_max,max_^6^	0.32	0.54	0.64	0.58	<0.01	0.52
*WS*_ave,max_^6^	0.49	0.46	0.69	0.68	0.42	0.55
*WG*_max,max_^6^	0.35	0.55	0.54	0.59	0.19	0.44
*WG*_ave,max_^6^	0.55	0.56	0.72	0.71	0.45	0.60
*WS*_max,max_^7^	0.34	0.58	0.68	0.6	<0.01	0.55
*WS*_ave,max_^7^	0.55	0.46	0.71	0.72	0.43	0.57
*WG*_max,max_^7^	0.37	0.58	0.5	0.61	0.16	0.44
*WG*_ave,max_^7^	0.63	0.59	0.75	0.74	0.4	0.62
*WS*_max,max_^8^	0.35	0.6	0.69	0.62	<0.01	0.57
*WS*_ave,max_^8^	0.61	0.44	0.72	0.74	0.42	0.59
*WG*_max,max_^8^	0.38	0.6	0.46	0.62	0.12	0.44
*WG*_ave,max_^8^	0.69	0.61	0.76	0.77	0.45	0.66
*WS*_max,max_^9^	0.36	0.62	0.7	0.62	<0.01	0.58
*WS*_ave,max_^9^	0.67	0.42	0.71	0.75	0.41	0.59
*WG*_max,max_^9^	0.39	0.60	0.41	0.63	0.1	0.43
*WG*_ave,max_^9^	0.75	0.61	0.76	0.78	0.45	0.67
*WS*_max,max_^10^	0.36	0.63	0.7	0.63	<0.01	0.58
*WS*_ave,max_^10^	0.72	0.39	0.69	0.75	0.4	0.59
*WG*_max,max_^10^	0.39	0.6	0.36	0.63	0.07	0.41
*WG*_ave,max_^10^	0.80	0.61	0.75	0.78	0.44	0.68
*WS*_max,max_^11^	0.37	0.64	0.69	0.62	<0.01	0.58
*WS*_ave,max_^11^	0.77	0.36	0.67	0.75	0.39	0.59
*WG*_max,max_^11^	0.39	0.58	0.31	0.63	0.06	0.39
*WG*_ave,max_^11^	0.84	0.61	0.74	0.78	0.42	0.68
*WS*_max,max_^12^	0.37	0.64	0.68	0.62	<0.01	0.58
*WS*_ave,max_^12^	0.81	0.33	0.65	0.74	0.38	0.58
*WG*_max,max_^12^	0.39	0.57	0.27	0.62	0.05	0.38
*WG*_ave,max_^12^	0.87	0.6	0.73	0.77	0.41	0.68
*WS*_max,max_^13^	0.36	0.63	0.67	0.62	<0.01	0.57
*WS*_ave,max_^13^	0.84	0.30	0.63	0.73	0.37	0.57
*WG*_max,max_^13^	0.38	0.55	0.24	0.62	0.04	0.37
*WG*_ave,max_^13^	0.89	0.59	0.72	0.77	0.4	0.67
*WS*_max,max_^14^	0.36	0.63	0.66	0.61	<0.01	0.57
*WS*_ave,max_^14^	0.86	0.27	0.6	0.73	0.36	0.56
*WG*_max,max_^14^	0.37	0.53	0.21	0.62	0.03	0.35
*WG*_ave,max_^14^	0.91	0.58	0.71	0.76	0.39	0.67
*WS*_max,max_^15^	0.35	0.62	0.65	0.61	<0.01	0.56
*WS*_ave,max_^15^	0.88	0.24	0.58	0.72	0.35	0.55
*WG*_max,max_^15^	0.36	0.51	0.18	0.61	0.02	0.34
*WG*_ave,max_^15^	0.92	0.57	0.69	0.75	0.39	0.66
*WS*_max,max_^20^	0.32	0.59	0.61	0.58	<0.01	0.42
*WS*_ave,max_^20^	0.93	0.15	0.49	0.68	0.31	0.51
*WG*_max,max_^20^	0.3	0.43	0.1	0.59	0.01	0.29
*WG*_ave,max_^20^	0.95	0.50	0.64	0.72	0.36	0.63

**Fig. A.2:**
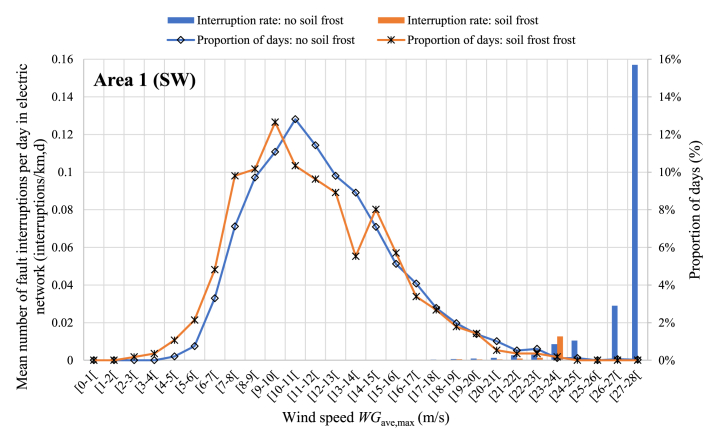
Interruptions caused by wind in Area 1(SW). The bars show the mean number of interruptions, and the line diagram depicts the proportion of days with the wind speed *WG*_ave,max_, which is the daily maximum of average hourly measured maximum wind gusts. The data contain 3457 days without soil frost and 561 days with soil frost. The total number of interruptions in the electricity distribution network was 21 979 without soil frost and 1273 with soil frost.

**Fig. A.3:**
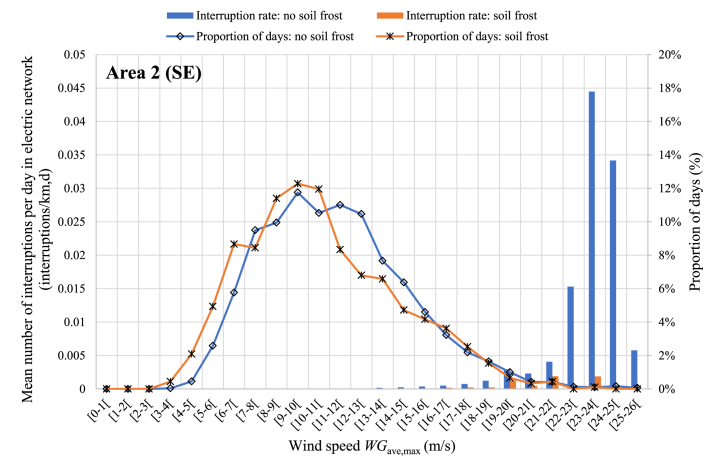
Interruptions caused by wind in Area 2(SE). The bars show the mean number of interruptions, and the line diagram depicts the proportion of days with the wind speed *WG*_ave,max_, which is the daily maximum of average hourly measured maximum wind gusts. The data contain 3106 days without soil frost and 912 days with soil frost. The total number of interruptions in the electricity distribution network was 8649 without soil frost and 413 with soil frost.

**Fig. A.4:**
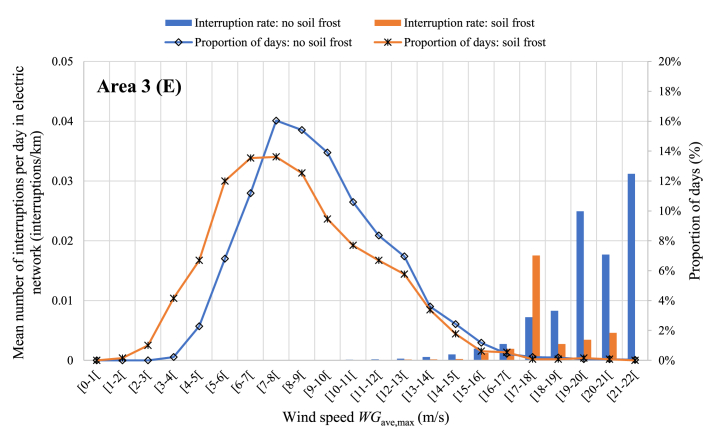
Interruptions caused by wind in Area 3(E). The bars show the mean number of interruptions, and the line diagram depicts the proportion of days with the wind speed *WG*_ave,max_, which is the daily maximum of average hourly measured maximum wind gusts. The data contain 2718 days without soil frost and 1300 days with soil frost. The total number of interruptions in the electricity distribution network was 21 784 without soil frost and 3628 with soil frost.

**Fig. A.5:**
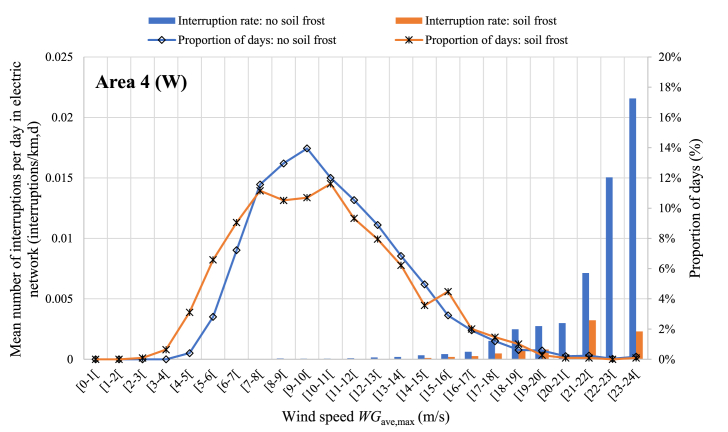
Interruptions caused by wind in Area 4(W). The bars show the mean number of interruptions and the line diagram depicts the proportion of days with the wind speed *WG*_ave,max_, which is the daily maximum of average hourly measured maximum wind gusts. The data contain 2924 days without soil frost and 1094 days with soil frost. The total number of interruptions in the electricity distribution network was 20 984 without soil frost and 1897 with soil frost.

**Fig. A.6:**
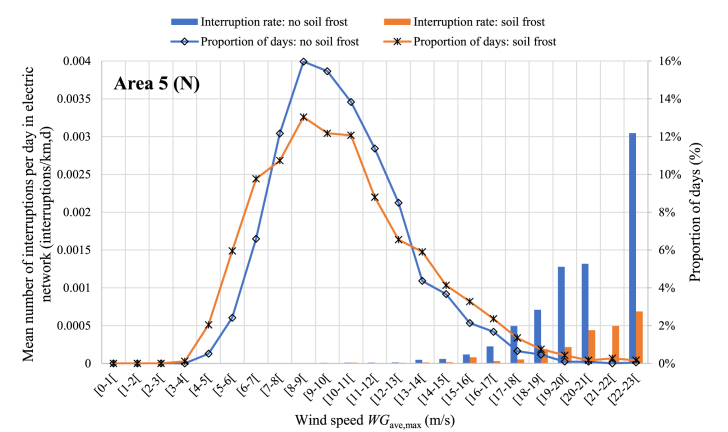
Interruptions caused by wind in Area 5(N). The bars show the mean number of interruptions, and the line diagram depicts the proportion of days with the wind speed *WG*_ave,max_, which is the daily maximum of average hourly measured maximum wind gusts. The data contain 2154 days without soil frost and 1864 days with soil frost. The total number of interruptions in the electricity distribution network was 685 without soil frost and 313 with soil frost.
